# Effect of Superovulation Treatment on Oocyte’s DNA Methylation

**DOI:** 10.3390/ijms232416158

**Published:** 2022-12-18

**Authors:** Jordana S. Lopes, Elena Ivanova, Salvador Ruiz, Simon Andrews, Gavin Kelsey, Pilar Coy

**Affiliations:** 1 Physiology of Reproduction Group, Department of Physiology, Faculty of Veterinary, University of Murcia, 30100 Murcia, Spain; 2 Institute for Biomedical Research of Murcia, IMIB-Arrixaca, 30100 Murcia, Spain; 3 Epigenetics Programme, The Babraham Institute, Cambridge CB22 3AT, UK; 4 Bioinformatics Group, The Babraham Institute, Cambridge CB22 3AT, UK; 5 Centre for Trophoblast Research, University of Cambridge, Cambridge CB2 3EG, UK

**Keywords:** oocyte, DNA methylation, ovarian stimulation, ART, epigenetics

## Abstract

Controlled ovarian stimulation is a necessary step in some assisted reproductive procedures allowing a higher collection of female gametes. However, consequences of this stimulation for the gamete or the offspring have been shown in several mammals. Most studies used comparisons between oocytes from different donors, which may contribute to different responses. In this work, we use the bovine model in which each animal serves as its own control. DNA methylation profiles were obtained by single-cell whole-genome bisulfite sequencing of oocytes from pre-ovulatory unstimulated follicles compared to oocytes from stimulated follicles. Results show that the global percentage of methylation was similar between groups, but the percentage of methylation was lower for non-stimulated oocytes in the imprinted genes *APEG3*, *MEG3*, and *MEG9* and higher in *TSSC4* when compared to stimulated oocytes. Differences were also found in CGI of imprinted genes: higher methylation was found among non-stimulated oocytes in *MEST* (*PEG1*), *IGF2R*, *GNAS (SCG6)*, *KvDMR1 ICR UMD*, and *IGF2*. In another region around *IGF2*, the methylation percentage was lower for non-stimulated oocytes when compared to stimulated oocytes. Data drawn from this study might help to understand the molecular reasons for the appearance of certain syndromes in assisted reproductive technologies-derived offspring.

## 1. Introduction

Assisted reproductive technologies (ART) have been clinically used for more than a century: from animals to humans, their use is now widely spread around the globe and the improvements seen over the years are enormous [1,2]. Parallel to developments in ART, studies made to investigate their possible consequences on the short- and long-term health of offspring are increasing, and now, with the advance of laboratory techniques (particularly the evolution of the epigenome analysis methodologies), flaws that were previously undetected are beginning to emerge [3].

Controlled ovarian stimulation (COS) is a necessary step in many ART procedures as it allows the collection of female gametes at a precise time and in suitable numbers for subsequent in vitro fertilization (IVF) or intracytoplasmic sperm injection (ICSI). The response to this ovarian stimulation is individual dependent, being conditioned by several factors such as type of hormones used, dose and timing of the treatment, body mass index, reproductive history, ovarian reserve, age, or even genetics [4]. Current protocols used in human fertility clinics are more individual-focused and include a pre-estimation of the functional ovarian reserve before deciding the type of hormonal administration. The use of a Gonadotropin-releasing hormone (GnRH) agonist or antagonist, as well as the daily dosage of Follicle-stimulating hormone (FSH) and the number of days of treatment, may vary between patients [1,5].

Although ovarian stimulation continues to evolve as a procedure in ART, the consequences of the release of this amount of exogenous hormone into the bloodstream remain insufficiently understood. Compromises of several aspects of oocyte structure or endometrial receptivity have been described [6]. Studies in mice [7] have shown that embryos from superovulated donors have retarded development in vitro; plus, embryo transfer to stimulated recipients revealed impaired implantation rate, higher mortality, and decreased fetal weight. In humans, a recent retrospective study [8] associated the high levels of estrogen found at the time of embryo transfer (ET) with higher levels of miscarriage and diminished live births per transfer when comparing fresh to frozen-thawed ICSI-derived embryos. Another retrospective study [9] identified the risk of lower birth weight when conceived through a cycle of COS + IVF + ET versus ET of a frozen-thawed embryo. A further concern regarding the use of exogenous hormones is the increased risk of developing ovarian hyperstimulation syndrome [10].

There could be multiple factors that contribute to any detrimental effects of hormonal stimulation on oocyte quality. One factor with the potential to have long-lasting outcomes in the embryo and offspring is the alteration in epigenetic information, in particular, because correct DNA methylation acquisition at imprinted genes is essential for their correct regulation in offspring (reviewed by [3,11]). This underlies the concern that ART might elevate the risk of imprinted gene-related syndromes [12,13,14]. Studies in mouse oocytes have indicated altered expression of genes involved in de novo DNA methylation in response to hormonal stimulation [15], and individual gene analysis in human oocytes has described altered methylation of imprinted genes [16]. On the other hand, it has also been reported that methylation errors detected at imprinted genes in mouse blastocysts after superovulation of oocytes do not arise in oocytes, but instead reflect impaired maintenance of methylation during preimplantation development [17]. Genome-wide profiling of DNA methylation in mouse oocytes also indicated very limited, if any, abnormalities associated with superovulation, at least in adult females [18]. Besides the altered methylation pattern that superovulated oocytes might exhibit, many others have also identified changes in gene expression associated with the hormonal stimulation [15,16,19].

Though the majority of studies seeking to evaluate the effects of ovarian stimulation have employed a rodent model, the bovine species represents an excellent alternative to study reproductive issues since cows are mono-ovulatory with a continuous cycle and carry primarily singleton pregnancies with a gestation period of nine months, all characteristics shared with humans. Additionally, the similarity in the amino acid sequence of most proteins is more conserved between cattle and humans than between mice and humans, as well as the chromosomal organization [20], and the fact that the complete genome sequence of cattle is now available, highlight the suitability of using bovine as a human-study model. The procedure of COS followed by transvaginal aspiration is also commonly used in the cattle industry [21], making the cow not only a valuable model for the study of oocytes [22] but also contributing to reduce and refine the number of animals used in animal research (3Rs).

Despite the advantages of the bovine model outlined above, a challenge is that the species is not as genetically inbred as rodent models, leading to sources of individual variation that may influence the response to hormonal stimulation both at the physiological level as well as genetically. The same challenge applies to human material. To account for such variation, in this study we used the same individual animal to collect both non-hormone-stimulated and hormone-stimulated oocytes. We then applied cutting-edge single-cell bisulphite sequencing to compare the DNA methylation profile, genome wide, of oocytes from the same animals with and without ovarian stimulation as a definitive test of the effect of this procedure.

## 2. Results

### 2.1. Global Methylation Profile of Oocytes from Ovarian-Stimulated versus Non-Stimulated Cows

In a first approach, and after the quality assessment, data from bovine oocytes were merged for analysis. The sequencing output of all libraries is given in Supplementary Table S1. From this approach, we found that 72.97% of genomic CG occurrences were represented for the S group, 24.12% for the NS group, 49.61% for Cow1, 35.43% for Cow2, and 35.18% for Cow3. 

To obtain an unbiased view of the global DNA methylation landscape of the cow in vivo matured oocytes, we defined tiles of 500 CGs through the Bos Taurus UMD 3.1 v91 genome assembly, and the tiles (N = 41,095) were quantitated to obtain their methylation percentages provided they contained at least 5 separate CG observations per tile. The quantitative percentage methylation of cow oocytes from non-stimulated and stimulated cycles as well as the percentage from each animal are presented in Table 1.

**Figure 1:**
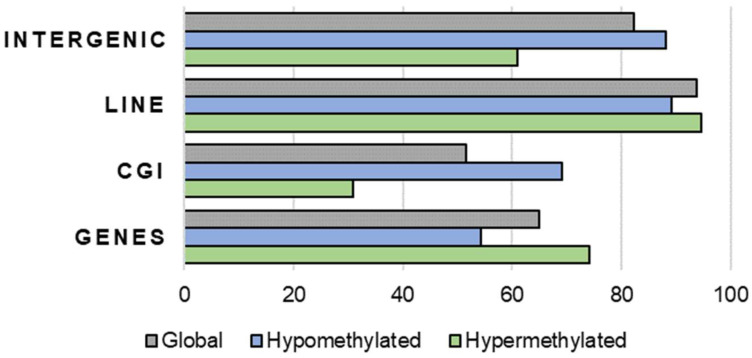
Proportions of different genomic features overlapping DNA hypermethylated (colour green) and hypomethylated (colour blue) tiles in cow oocytes compared to the whole genome (global, colour grey).

Under the above-mentioned conditions, we observed 11,163 hypermethylated tiles (>75% methylation), representing 25.5% of the total tiles, and 8193 hypomethylated tiles (<25% methylation), representing 18.75% of the total tiles. Those tiles were distributed among the different genomic features as expected (i.e., CGI and intergenic regions were mainly hypomethylated, and genes and LINE mainly hypermethylated), supporting the validity of our results overall (Figure 1).

### 2.2. Identification of Differentially Methylated Regions (DMRs) in Oocytes from Ovarian Stimulated and Non-Stimulated Animals

To find differentially methylated regions (DMR) in oocytes from ovarian S and NS animals, we performed a global unbiased (Section 2.2.1) and targeted analysis of the sequenced data (Section 2.2.2), as well as a third analysis focused on methylation of imprinted genes (Section 2.2.3). The unbiased analysis was done under the same conditions used for the description of the global methylation profile of the oocytes. The targeted analysis was performed by splitting the cow genome in hypermethylated and hypomethylated domains and under more restrictive conditions (100 CG tiles and at least 10 CG separate sites per tile). 

#### 2.2.1. Unbiased Analysis of DNA Methylation in Oocytes for Ovarian Stimulated versus Non-Stimulated Animals

**Figure 2:**
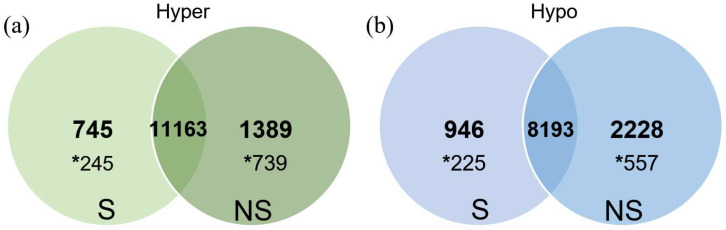
Venn diagram of hypermethylated (**a**) and hypomethylated (**b**) tiles from stimulated (S) and non-stimulated (NS) oocytes, exhibiting the number of exclusive tiles hyper (**a**) or hypo (**b**) methylated in each group and the common tiles hyper (**a**) or hypo (**b**) methylated between the groups. Within each group, the number preceded by an asterisk represents the number of tiles with a minimum of 10% absolute change in methylation percentage between groups (*p* < 0.05).

For the comparison between the S versus the NS animals, we decided to merge all the single-cell libraries for each treatment into one only dataset to maximise the number of observations we had to work with, and therefore the statistical power for the test. Doing so, we found a total of 11,908 and 12,552 total hypermethylated tiles, for each group (S vs NS), respectively, and 11,163 were common between both groups (Figure 2a). By filtering only the tiles with a minimum of 10% absolute change in methylation, we found a total of 245 hypermethylated tiles in the S group compared to the NS group (*p* < 0.05) and 739 hypermethylated tiles in the NS group compared to the S group (Supplementary Table S2). 

Regarding the hypomethylated tiles (Figure 2b), we found 9139 for the S group versus 10,421 for the NS group. Again, from them, 8193 were common tiles for both groups, whereas 946 were present only in the S group (225 of them with more than 10% absolute change in methylation between groups, *p* < 0.05) and 2228 only in the NS group (557 with more than 10% of methylation difference, *p* < 0.05, Supplementary Table S2).

Amongst the DMRs hypermethylated in the S animals, a tile overlapping *DNMT1* and including a CGI at the promoter region was found, with a methylation percentage of 78.95%, significantly different from the NS animals where the percentage was 62.3%.

**Figure 3:**
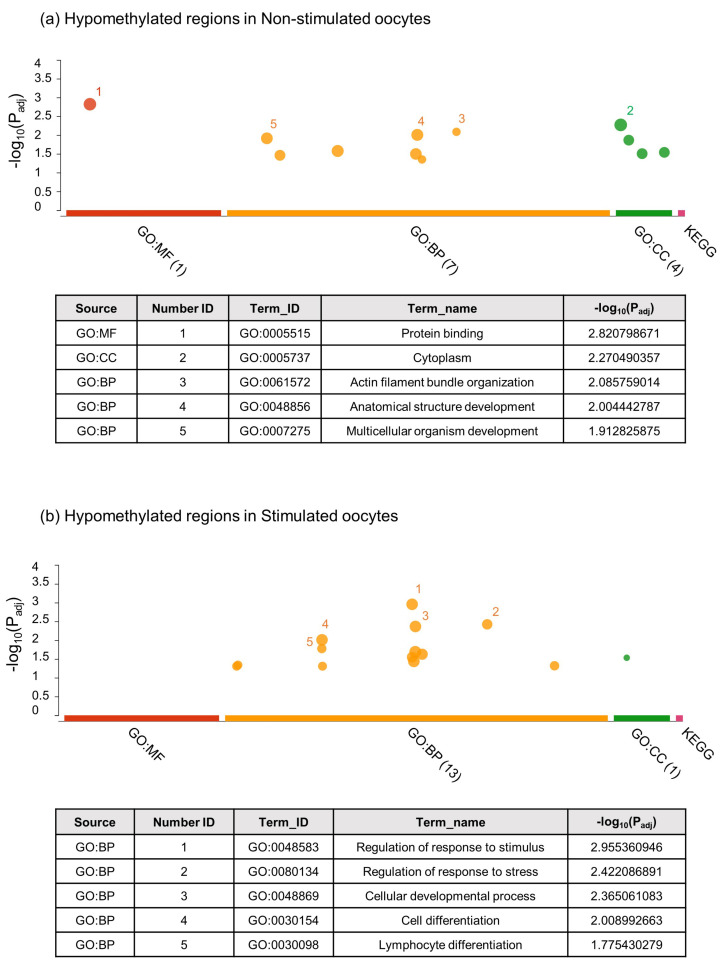
Manhattan plot of g:Profiler enrichment results of hypomethylated DMRs obtained in non-stimulated (**a**) and stimulated oocytes (**b**). GO (BP = biological process (orange colour); MF = molecular function (red colour); and CC = cellular component (green colour)) and KEGG database (pink colour) were used for the analysis. Only terms with a *p* value < 0.05 are shown. The table below shows the top five enriched representative terms, and the statistical value for each term is indicated by the -Log10 (*p* value).

**Figure 4:**
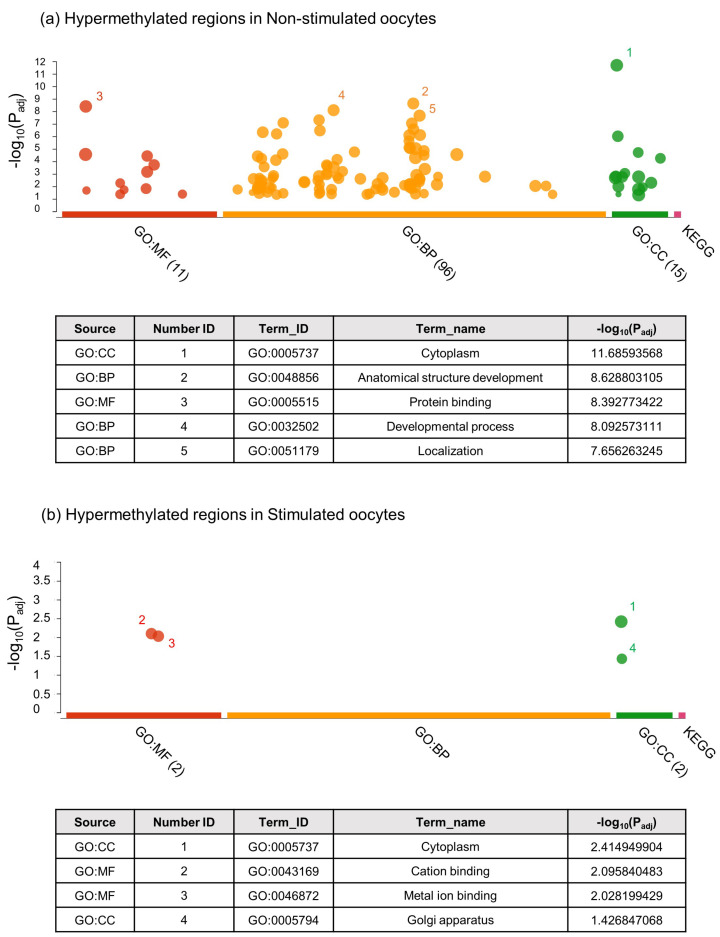
Manhattan plot of g:Profiler enrichment results of hypermethylated DMRs obtained in non-stimulated (**a**) and stimulated oocytes (**b**). GO (Molecular Function – MF, red colour; Biological Process – BP, orange colour; and Cellular Component – CC, green colour) and KEGG database (pink colour) were used for the analysis. Only terms with a *p* value < 0.05 are shown. The table below shows the top five enriched representative terms, and the statistical value for each term is indicated by the −Log10 (*p* value).

The g:Profiler [23] was used to perform the functional enrichment analysis of the DMRs to better understand the functions and metabolic pathways. Supplementary Table S3 describes all the obtained results for hypo and hypermethylated DMRs in both groups. Figure 3a shows the five most overrepresented processes for hypomethylated regions in NS oocytes, which were: GO:005515—Protein binding; GO:005737—Cytoplasm; GO:0061572—Actin filament bundle organization; GO:0048856—Anatomical structure development; and GO:007275—Multicellular organism development. In S oocytes (Figure 3b), the five most overrepresented hypomethylated processes were: GO:0048583—Regulation of response to stimulus; GO:0080134—Regulation of response to stress; GO:0048869—Cellular developmental process; GO:0030154—Cell differentiation; and GO:0030098—Lymphocyte differentiation. Figure 4a shows the functional enrichment analysis for hypermethylated regions in NS oocytes, where the most overrepresented terms were: GO:0005737—Cytoplasm; GO:0048856—Anatomical structure development; GO:0005515—Protein binding, GO:0032502—Developmental process; and GO:0051179—Localization. In S oocytes (Figure 4b), the most overrepresented hypermethylated processes were: GO:0005737—Cytoplasm; GO:0043169—Cation binding; GO:0046872—Metal ion binding; and GO:0005794—Golgi apparatus.

#### 2.2.2. Targeted Analysis of Cow Oocyte DNA Methylation by Segmenting the Genome in Hypermethylated and Hypomethylated Domains for two Treatments (S versus NS animals)

**Figure 5:**
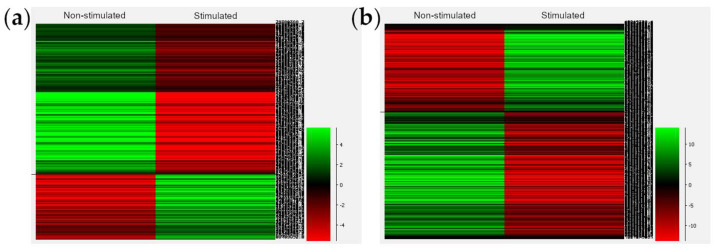
(**a**) Heatmap for oocyte 256 DMRs between oocytes from non-stimulated and stimulated cows using the 21 best-covered libraries and considering the animals as biological replicates after segmenting the genome in methylated and unmethylated domains; (**b**) Heatmap for oocyte 5220 DMRs between non-stimulated and stimulated cows using the merged data sets from the three animals in one single biological replicate after segmenting the genome in methylated and unmethylated domains. On the right side of each heatmap, the scale represents the methylation status, where green corresponds to a lower methylation and red to a higher methylation.

In a second attempt to identify DMRs between treatments, a targeted and more restrictive analysis was performed by segmenting the whole cow genome into contiguous methylated and unmethylated domains. Data from all samples with high coverage were combined and quantitated for percentage methylation, using tile sizes of 100-CGs. Adjacent tiles were then merged if they retained the same state as the previous tile, either hypermethylated (>50%) or hypomethylated (<50%). The merged domains were then used to quantitate percentage methylation in all of the individual cells. A total of 15,123 methylated domains and 15,954 unmethylated domains were detected (Supplementary Table S4), and the methylation was quantitated requiring at least 10 observed individual CG sites per domain to be included in the analysis. Doing so, we identified a total of 3645 tiles measured in all the libraries with a total of 256 DMRs statistically significant (corrected *p* < 0.05) based on a comparison of the biological replicates of the S and NS samples. The representative heatmap for these DMRs is shown in Figure 5a. 

When the single-cell libraries were merged and analysed as two unique datasets from S and NS animals over the same probes (methylated and unmethylated domains), we found 5220 DMRs (*p* < 0.05). The representative heatmap is shown in Figure 5b.

**Figure 6:**
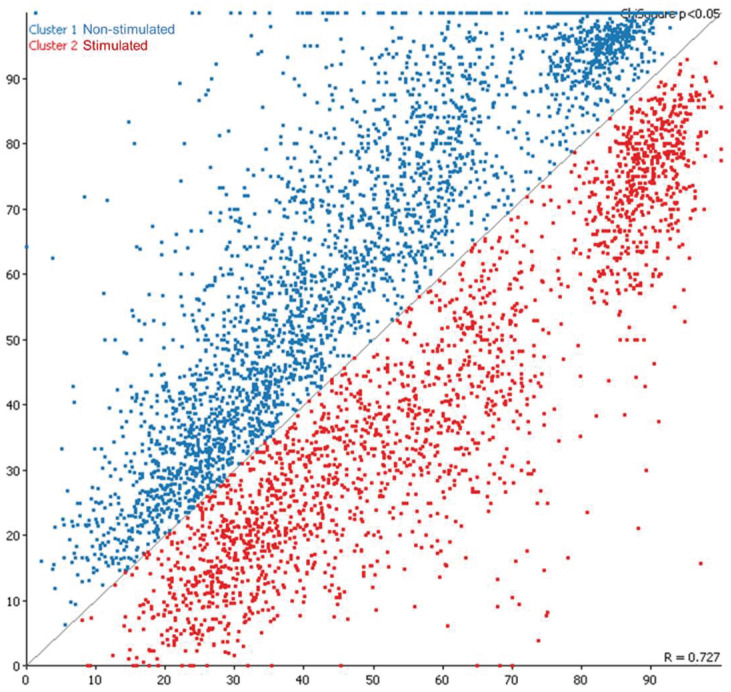
Scatter plot for oocyte 5220 DMRs between non-stimulated and stimulated cows.

The corresponding annotation for the DMRs with the merged (5220) and unmerged (256) datasets are shown in Supplementary Table S5. The representative scatter plot on the variation of methylated and unmethylated regions between S and NS animals is shown in Figure 6.

Amongst the DMRs identified in the merged analysis, we found a domain overlapping another member of the *DNMT* family, *DNMT3B*, with significantly different methylation percentages of 71.25% for the S group and 79.43% for the NS group. In addition, differences were found in tiles overlapping two genes for methyl CG binding proteins, *MBD1* (44.86% vs. 54.87%) and *MBD3L1* (33.37% vs. 19.57 in S vs. NS).

**Figure 7:**
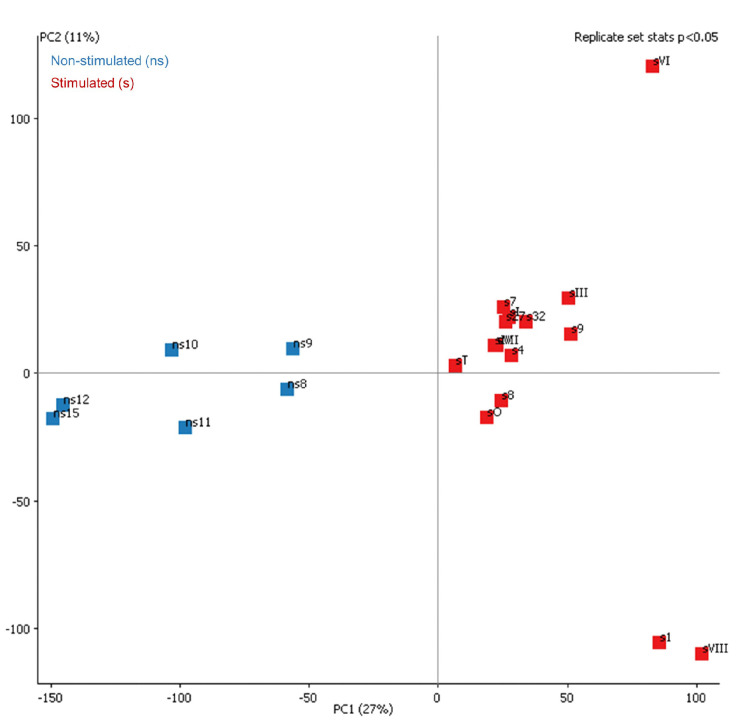
Principal component analysis for the 256 oocyte DMRs between a selected number of samples with higher coverage of non-stimulated and stimulated animals.

With the unmerged approach, we obtained a significant number of DMRs as corroborated by the principal component analysis shown in Figure 7.

**Figure 8:**
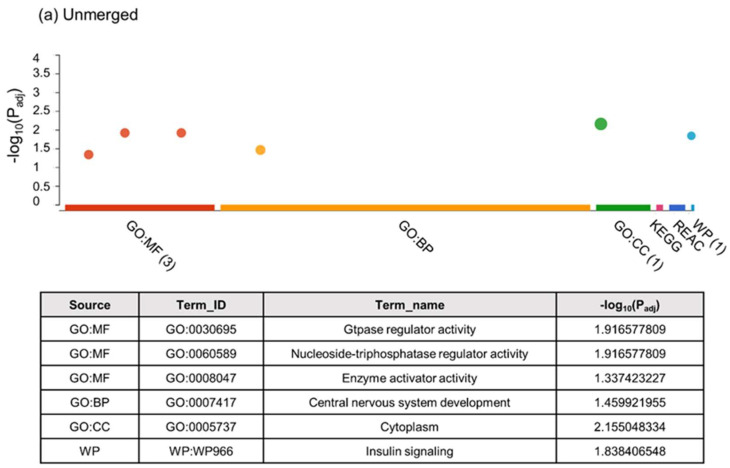

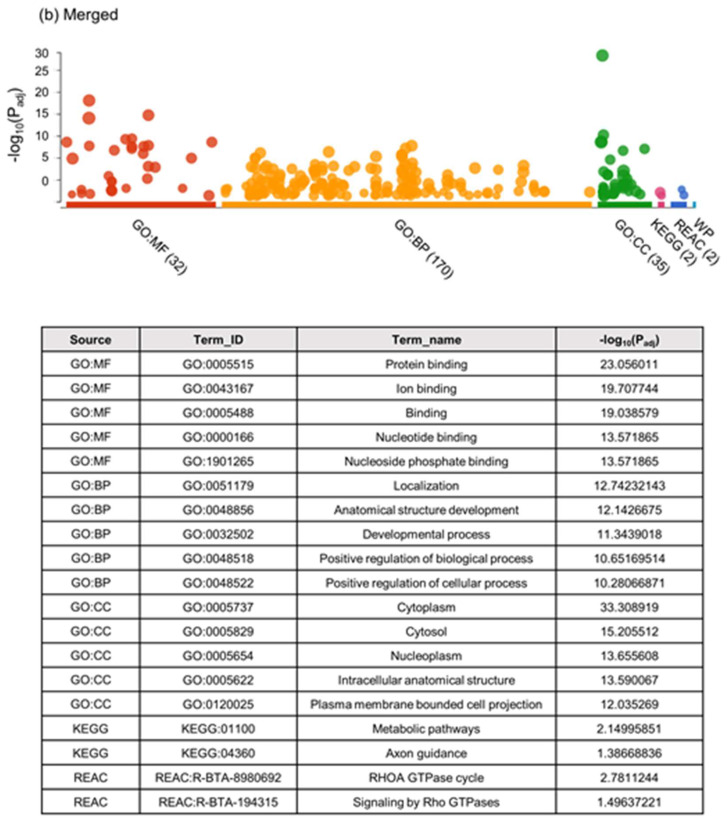
Functional enrichment analysis with g:Profiler of DMRs obtained by segmenting the genome in hypermethylated and hypomethylated domains for two treatments (stimulated and non-stimulated animals). Manhattan plot of g:Profiler enrichment results obtained for unmerged (**a**) and merged (**b**) conditions. GO (Molecular Function – MF, red colour; Biological Process – BP, orange colour; and Cellular Component – CC, green colour), KEGG (pink colour), Reactome (navy blue colour), and WikiPathways (light blue colour) databases were used for the analysis. Only terms with a *p* value < 0.05 are shown in the graph. The table below shows the most representative terms for each analysis group (where applicable), and the statistical value for each term is indicated by the −Log_10_ (*p* value).

The g:Profiler was used, together with Reactome and WikiPathways databases, to evaluate the functional enrichment analysis of the DMRs. The analysis was made using the merged and unmerged approach described previously, and all the obtained results are described in detail in Supplementary Table S6. Figure 8a shows the overrepresented processes using the unmerged strategy. Different cellular processes such as GTPase regulator activity, Nucleoside-triphosphatase regulator activity, or central nervous system development are overrepresented in the DMRs. In addition, an important signalling pathway such as insulin signalling is also affected by the methylation process. Using the merged strategy, we were able to distinguish a greater number of processes, since the analysis introduced a greater number of methylated regions. Figure 8b shows the overrepresented processes for GO (Molecular Funtion (MF), Biological Processes (BP), Cellular Component (CC)), KEGG, Reactome, and WikiPahtways (where applicable). The analysis showed how binding-related processes are overrepresented in the MF group. The BP analysis revealed how the methylation process affects cellular processes related to anatomical structure and developmental processes. The KEGG analysis identified pathways related to metabolism and axon guidance whereas Reactome identified signalling pathways related to GTPase signalling.

#### 2.2.3. Targeted Analysis of Cow Oocyte DNA Methylation against Imprinted Genes for Two Treatments (Ovarian S versus NS Animals)

A final analysis was done against specific genes of interest taken from the literature, namely the *DNMT* family and the imprinting genes in the cow. The methylation percentages at genes for the two groups of oocytes is shown in Supplementary Table S7, and the list of genes with significant differences is shown in Table 2. The methylation percentages at CGI for the two groups of oocytes is represented in Supplementary Table S7, and the corresponding statistical significance after *χ*^2^ analysis are shown in Table 3. Our data showed significant differences in the methylation levels between both groups for *APEG3*, *MEG3*, *MEG9*, and *TSSC4*. For the first three genes, we observed a gain of methylation in S animals whereas a loss of methylation was detected for *TSSC4*. In addition, *APEG3* was hypomethylated in our NS animals compared to the S group, although *APEG3* CGI was practically unmethylated in both groups.

## 3. Discussion

In this study, we have generated for the first time, to the best of our knowledge, the methylome of cow oocytes coming from the same animals before and after an ovarian stimulation treatment. Global methylation was found to be quite similar between S and NS oocytes. Nonetheless, differences were found in specific imprinted genes.

One of the limitations of our study was detected after performing the quality control of our samples. We detected contamination from somatic cells and reduced the number of samples to those that did not exhibit contamination and had the best coverage. Even though our initial goal was to compare individually the effect of the stimulation treatment, we were still able to compare the effect of the treatment by grouping the three different animals. This is also a plus of using the bovine as a model, since with this animal we are able to perform transvaginal oocyte aspiration and still keep the same animal, reducing the number of animals used. However, collecting a single oocyte from the NS cycles was a challenge. The transvaginal follicle aspiration is a technique that requires expensive equipment, a skilful technician, and good control over the oestrus cycle. In the present study, a period of two years was necessary to collect all the samples. The rate of success per session can reach 70% when multiple follicles are aspirated from stimulated ovaries [24]. However, with animals under natural cycles and in absence of hormonal stimulation, it is necessary to continuously monitor the follicles to perform the aspiration of the dominant preovulatory follicle, typically reaching >15 mm in bovine species [25]. The recovery rate from pre-ovulatory follicles (NS group) after transvaginal aspiration was around 38%, which was an expected limitation since, as reported in humans (reviewed by [1]), aspiration of single oocytes shows limited success.

As for the difference in methylation patterns due to the different ages of the cows, it is still a matter of debate. Castillo-Fernandez et al. [26], using mice oocytes, found that CG methylation was reduced in aged females. Nonetheless, the bimodal methylation landscape of the oocyte was maintained regardless of age as well as methylation of DMRs in the germline. Another study in mice [27] showed that it is not about the age of the oocyte, but rather the treatment it receives that influences the methylation levels later at the embryonic stage. In our study, we drove our focus on the advantage of obtaining oocytes with and without hormonal stimulation from the same exact animals, since the cow is a suitable animal model for this type of necessity. We cannot exclude that the age of the cows may play a role in the methylation patterns, nor can we conclude that it does based on our low number of animals.

Previous studies looking for differences in phenotypes between embryos and fetuses derived from S and NS animals have shown alterations in developmental dynamics, implantation, fetuses’ weight, pre and perinatal mortality rates, or skeletal ossification [7,9,28,29,30]. Adverse effects of COS treatments in already conceived children such as increased incidence of musculoskeletal defects [31] and higher long-term medication use and hospital-care episodes have been also described ([32]; reviewed by [33]). To explain these findings, it has been proposed that COS treatments alter oocyte competence, probably by preventing the proper reprogramming of epigenetic marks [34,35,36,37] and, consequently, altering gene expression. However, this hypothesis has not ever been demonstrated.

Indeed, studies at a molecular level, including differences in oocyte transcriptome and methylome, are scarcer and sometimes contradictory. Chu et al. [19] showed an increase in mRNA levels of *BTG4*, *PTTG1*, *PAPOLA*, and *LEO1* in cow oocytes from animals treated with FSH, and Uysal et al. [15] found that *DNMT1*, *DNMT3A*, and *DNMT3B* protein expression in mouse oocytes is also affected by ovarian stimulation treatments. While from our data we have not been able to corroborate differences in methylation for *BTG4*, *PTTG1*, *PAPOLA*, and *LEO1*, we did find differences in a tile overlapping *DNMT3b* that showed a loss of methylation in the oocytes from the S compared to the NS animals. These data do not necessarily correlate with the lower *DNMT3B* expression found by Usyal et al. in their S group of oocytes according to the expected correlation between methylation levels at gene bodies and gene expression, although that correlation seems to be not always happening [38]. We also found in our unbiased non-targeted approach, that a DMR overlapping *DNMT1* was significantly more methylated in S animals than in NS ones. This piece of evidence would suggest that both re-methylation and de novo methylation activities are altered in the S oocytes compared to the non-treated ones, but the short-term scope of these findings cannot be inferred from the present study. It is noteworthy to mention that during folliculogenesis, transcriptional quiescence is to be expected, and other authors have shown that the use of gonadotropins to stimulate follicle growth may alter this inactivity by increasing the proportion of transcriptionally inactive oocytes [39]. The possible role of *DNMT1* in this process should be investigated in the future.

In addition, we also found differences under this same approach in tiles overlapping two methyl-CG binding proteins, *MBD1* (44.86% vs. 54.87%) and *MBD3L1* (33.37% vs. 19.57% in S vs. NS), whose physiological significance remains to be corroborated since previous reports had shown differences at blastocyst but not at oocyte level in the expression of these genes [33].

As for global DNA methylation, studies have shown differences in 10–20% of methylation percentages of 5MeC, measured via immunofluorescence, between mouse embryos from S females compared to NS [40], whereas Huffman et al. [34] found a 50% reduction in global DNA methylation (assessed by measuring the level of acetylated H3K9/14), exclusively on the maternal genome of zygotes. In addition, contradictory data indicating failures in oocyte DNA methylation after hormone stimulation [35] or the lack of differences have been also reported [41,42]. From our results, we cannot conclude that the hormone stimulation treatment induces differences higher than 2% in the global methylation levels of cow oocytes, and this fact is, on the other hand, the most plausible result expected, since the success of live births in humans and animals after COS treatments is already showing that the expected consequences, if any, should be related to specific genome features and hardly could be expected as affecting the whole genome.

Regarding specific DMRs, Sato et al., in 2007 [16], found that *H19* DMR, which should be unmethylated in the maternal germline, showed methylation in humans and mice oocytes. However, from our data, we could not see a difference in the methylation level at *H19* or at *H19/IGF2* ICR, which remained unmethylated in both S and NS groups of oocytes. Other DMRs previously detected in oocytes or early embryos and mainly related to imprinting genes include *PEG3, KvDMR1, Snrpn*, and *IGFR2*, although the same genes have been reported as no-differentially methylated in other studies (reviewed by [33]). From our results, neither *PEG3* nor *IGF2R* or *Snrpn* were differentially methylated between S and NS animals, while not enough reads were obtained for *KvDMR1* in NS animals to compare with S animals.

*APEG3*, an antisense transcript gene harboured at the *PEG3* locus and displaying paternal allele-specific expression, was detected with a methylation level near hypomethylation (considered at 25% methylation) in our NS animals. More than 50% methylation was seen for this gene in the S animals, suggesting a possible loss of imprinting derived from the treatment. Our data over *APEG3* CGI, by contrast, did not corroborate such a hypothesis because no different methylation was detected for the closest CGI annotated to this gene, which was practically unmethylated in both groups. Reduction of *APEG3* expression has been found in the fetal placenta of a bovine model of intrauterine growth restriction [43], but no references have been found mentioning the consequences derived from a possible excess of *APEG3* expression, which theoretically would occur if the closest CGI were the ICR for this gene.

*MEG9*, maternally expressed gene 9 (paternally imprinted) in the bovine placenta, exhibited hypermethylation for the S group at the gene body but not for the NS with a 30% difference in the respective methylation percentages. We had to quantitate this gene in our study by including it manually in our annotation track, and the closest CGI was not automatically detected. Yet the corresponding methylation level for this CGI was 98.15% for the NS group and 67.6% for the S group, which could indicate a loss of methylation at the putative ICR (and the corresponding increase in gene expression) for the hormone-treated animals. This observation remains to be proven at the moment.

Another gene showing significant differences in methylation was *TSSC4*, again proposed as maternally expressed and paternally imprinted and belonging to the *Cdkn1c/Kcnq1ot1* imprinting cluster. In this case, the corresponding CGI was unmethylated in both groups without differences, corroborating the permissive status for the expression of the gene and thus making the interpretation of the methylation differences at the gene body difficult. In addition, this gene has been detected as showing biallelic expression in adults [44], complicating even further the obtaining of consistent conclusions.

Probably the most significant finding of the present study is the list of CGI differentially methylated between our two groups of oocytes because it includes two at the IGF2R gene and two at the *IGF2* gene, plus one at *PEG1*, one at *GNAS*, and the ICR *KvDMR1*. All of these features have been previously related to large offspring syndrome, the main aberration described in ART-derived offspring, not only in cattle but also in the similar Beckwith–Wiedemann syndrome in humans [12]. Consistent with this list, the global tendency, with one exception, was towards the loss of imprinting, represented in this case by the significant loss of methylation for all of them. *GNAS* has been also associated with oocyte quality [45], and it was found that its CGI was hypermethylated in superovulated oocytes from adult mice but hypomethylated in superovulated oocytes from prepubertal mice, those of presumed lesser quality. In our data, NS oocytes had higher methylation than those from S group, suggesting a better quality. Since the CG coverage for all these features varied between 15–60% in the data from NS animals and between 64–100% in those from S, we must conclude that, according to these results, the hypothesis that the oocyte could be the origin of the observed ART-derived alterations, and that the hormone stimulation could be the cause, should be accepted. Supporting this conclusion is the fact that, as shown by Ivanova et al. [46], the maternal genome undergoes a lower level of reprogramming after fertilization (methylation level drops from 60 to 45% approximately at the first division) compared to the paternal genome (methylation level drops from 80% to 45% at the 2 cell stage), and this percentage remains almost stable until the second drop at the blastocyst stage, where the minimum methylation level is reached (around 27%). This suggests that errors in the oocyte methylome, particularly at the imprinting genes, may not be completely corrected later in development and could remain in the offspring after birth and even until adult life, representing an explanation for the molecular mechanism of the ART-derived aberrations.

## 4. Materials and Methods

All chemicals were purchased from Sigma-Aldrich Chemical Company (Madrid, Spain), unless otherwise indicated. 

### 4.1. Ethics

The experimental work was submitted to evaluation by the CEEA (Comité Ético de Experimentación Animal) from University of Murcia. After approval, authorization from “Dirección General de Agricultura, Ganadería, Pesca y Acuicultura”—Región de Murcia- nr A13170706 was given to perform the animal experiments.

### 4.2. Animals

Non-lactating and non-pregnant cows, two Holstein (Cow1, birth date 17 March 2006, and Cow3, birth date 1 February 2015) and one Braunvieh (Cow2, birth date 14 May 2005), with ages between 1.5 and 11 years, were kept at the farm facilities at University of Murcia, Spain. Animals were used between October 2016 and July 2018.

### 4.3. Non-Stimulated (NS) Cows 

Cows were followed by ultrasound until the dominant follicle had the desired size for ovum pick up (≥15 mm of diameter, according to Pavlok et al. [25], as being the mean value to obtain oocytes resuming meiosis in vivo). At 5 days post-ovulation, prostaglandin (Dinolytic^®^, Zoetis, Madrid, Spain—5 mg/mL, 25 mg IM) was administrated in order to reduce estrous interval.

### 4.4. Hormone-Stimulated (S) Cows

Superovulation treatment consisted of administration of GnRH (Dalmarelin^®^, Fatro, Barcelona, Spain—25 mg/mL, 2 mL IM) and FSH/LH (Pluset, Calier, Barcelona, Spain—500 UI FSHp 500 IU LHp) as follows: time 0 h GnRH administration; 36 h, 3.5 mL of FSH/LH (IM) and 4 mL FSH/LH (SC) and 60 h, 2.5 mL of FSH/LH (IM). Transvaginal aspiration was performed at 96–98 h after first administration.

### 4.5. Transvaginal Oocyte Retrieval

Firstly, the cow was palpated, and ultrasound evaluation was performed in order to assess the size of follicle(s) or to count the number of follicles. The system used for follicle aspiration was a Falco-Vet ultrasound with a 10R transvaginal probe at 7.5 MHz (Esaote, Genova, Italy). Then, as a standard procedure, cows were given xylazine (Nerfasin^®^, Fatro, Barcelona, Spain—0.25 mL/100 kg weight, IM), carprofen (Carprosan^®^, Fatro, Barcelona, Spain—1.4 mg/kg weight, SC), and lidocaine (Anesvet, Ovejero, León, Spain—2%, 5 mL, epidural). For oocyte retrieval, the aspiration pump (Aspirator 3—Labotect, Göttingen, Germany) applied a pressure of 70 mm Hg, 20 mL/min, and the system included the punction needle (with a disposable 18 G needle) connected via a sterile tube to a Falcon tube. The medium used to collect oocytes was Dulbecco’s Phospate buffered saline, supplemented with 1% (v/v) foetal bovine serum and 2.2 UI/mL heparin, pre-heated at 38 °C.

### 4.6. Oocyte Retrieval and Storage

After aspiration, oocytes were collected under a stereomicroscope (Nikon SMZ 10 A, Tokyo, Japan) and washed twice in Dulbecco’s Phosphate buffered saline solution (without calcium nor magnesium, PBS) supplemented with 0.5% polyvinyl alcohol (wt/v). Cumulus cells were removed by gentle pipetting, and vortexing was used when necessary, as well as hyaluronidase (0.2% in PBS). Zona pellucida was removed using pronase (0.5% in PBS). Oocytes were put in 5 µL of RLT buffer (Qiagen, Hilden, Germany), immediately frozen in liquid nitrogen, and stored at −80 °C.

### 4.7. DNA Methylation: Single-Cell Methylome Analysis

An adaptation of whole genome bisulfite sequencing that involves post-bisulfite adaptor tagging (PBAT) to the single cell method was used to analyse the methylome of individual oocytes at single-base resolution on a genome-wide scale. The method was described in detail in Clark et al. [47]. Briefly, oocytes were lysed in 5 µL of RLT Plus buffer (Qiagen, Cat. No 1053393) for 10 min at room temperature, and bisulfite conversion was carried out directly on the cell lysate to minimise losses using the standard manufacture kit (Zymo Research, D5020). DNA was eluted in a first strand synthesis mix containing oligos with Illumina adaptor sequence at the 5′ end and the random nucleotide sequence at the 3′ end to facilitate binding in all genomic locations. Five rounds of pre-amplification were subsequently performed. Preamplification was followed by exonuclease I treatment to remove remaining oligos from the reaction mix, and a second strand-synthesis step was performed. Finally, libraries were purified with AMPure XP beads (Beckman Coulter, cat. no. A 63881) and amplified for 13 PCR cycles. After PCR amplification, purified libraries were subjected to quality control (QC) on a Bioanalyzer (Agilent 2100) and subsequently sequenced. A total of 15 libraries from NS animals (6 for Cow1, 4 for Cow2, and 5 for Cow3) and 30 from S animals (9 for Cow1, 6 for Cow2, and 15 for Cow3) were generated and prepared for 100 bp single-end sequencing on Illumina HiSeq 2000 and sequenced at 1/20 th of a lane per sample. Obtained data were processed using Bismark version 0.20.1 (https://www.bioinformatics.babraham.ac.uk/projects/bismark/, accessed on 21 November 2022) [48], and subsequent methylation calls were analysed using Seqmonk version 1.45.1. (https://www.bioinformatics.babraham.ac.uk/projects/seqmonk/, accessed on 21 November 2022).

This technique detects both 5-hmC and 5-mC methylation, and for this analysis, we assumed that the signal came from 5-mC since this is the major form.

For the enrichment analysis of DMRs, g:Profiler [23] was used. All genes in the genome proceeded from the enrichment background, using Bos taurus as reference genome. Only annotated genes were used. Terms with a *p* value < 0.05 according to g: SCS threshold were selected for further analysis. GO (Biological Process, Molecular Function, and Cellular Component), KEGG, Reactome, and WikiPathways were selected as reference databases. The terminology used to name the transcripts is that approved by the Ensembl. To eliminate redundant terms when the enrichment analysis provides a large number of terms, the Revigo [49] database was used.

### 4.8. Assessment of Data Quality

Bovine oocytes were processed for single-cell PBAT [47,50]. In order to check the quality of the data, the size of the 45 oocyte-sequenced libraries as well as the mapping efficiency were assessed. The efficiency was consistent across all samples although the absolute number of sequences differed (Supplementary Table S8). Then, three quality control (QC) filters were applied over the sequenced data. First, the overall amount of methylation in CG and non-CG contexts was evaluated in the 45 libraries in order to find out if some of the samples might be contaminated with non-oocyte DNA from somatic cells. As previously reported, it is expected to have reasonably high levels of non-CG methylation (~5%) in oocytes [51,52]. The analysis showed two fairly distinct groups of samples based on their CG (and to a lesser extent CH) methylation. The group with lower CH and higher CG methylation was classified as potentially contaminated since this inverted proportion over the expected one was not linked to any biological factor (neither S/NS animals nor to individual) (Supplementary Figure S1). A second QC analysis was based on the expectation that oocytes should have contiguous regions that show almost complete demethylation. We selected such an exemplar region on chromosome 5, which was about 1.3 Mbp in size and which was heavily demethylated in most of the samples, as expected in oocytes. After quantitating the methylation over this region, we found some samples with an increase in CG methylation (Supplementary Figure S2), and most of them correlated with the samples previously classified as potentially contaminated with non-oocyte DNA by their non-CG methylation level (Supplementary Figure S1). Finally, a third QC was performed based on methylation in CG islands (CGI) from the X chromosome (ChrX) based on the assumption that the X chromosome undergoes reactivation and demethylation during germline development, so it should have very low levels of CGI methylation in oocytes, whereas in female somatic cells with an inactivated ChrX CGI methylation should be ~50%, representing the mean of the active (unmethylated) and inactive (methylated) copies. We compared ChrX CGIs with those from chromosome 1 (Chr1), since it is the closest in size. After plotting the percentage of methylated/unmethylated calls over all the CGIs on ChrX and Chr1, we observed that most of the samples with >30% ChrX CGI methylation (Supplementary Figure S3) corresponded to those with an obvious deviation according to their methylation at the reference region (Supplementary Figure S2). Consequently, we discarded those samples from the study (n = 17) plus two others that were mislabelled and continued working with the 28 remaining libraries.

### 4.9. Statistics

The comparison of the percentage of methylation was performed using the *χ*^2^ test for the unbiased and targeted analysis and Student t-test followed by Benjamini–Hochberg correction for the segmented analysis. A corrected *p* value < 0.05 was considered statistically significant.

### 4.10. Experimental Design

**Figure 9:**
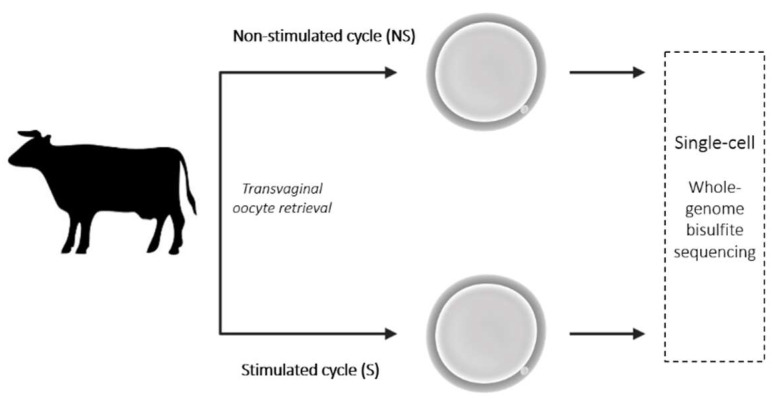
Schematic representation of the experimental design.

Non-stimulated cows with pre-ovulatory follicles were submitted to transvaginal aspiration (Figure 9). After obtaining the 4–6 oocytes per animal (Table 4), the same donor cows were submitted to hormonal stimulation and again subjected to transvaginal aspiration to collect samples. The procedure was repeated twice for each cow, with an interval between ovarian stimulation of above 3 months. Those samples were identified as derived from S animals (S). Oocytes were stored and processed individually and whole-genome bisulfite sequencing was performed.

## 5. Conclusions

In conclusion, in the present study, we have shown that the methylome of in vivo matured cow oocytes from NS and hormone S animals is globally similar, but, at the same time, we have been able to detect differences in the methylation levels of specific CGI of imprinted genes and ICR, indicating that the previously described alterations in ART offspring could have their origin in the hormonal treatment of the females at the beginning of the procedures.

## Figures and Tables

**Table 1:** Global mean DNA methylation percentages of single cell oocyte libraries grouped by treatment (non-stimulated and stimulated) and by animal (Cow 1, Cow 2, and Cow 3).

		**Global Mean Methylation (%)**
Treatment		
	Non-stimulated	52.91
	Stimulated	51.21
Animal		
	Cow 1	51.29
	Cow 2	53.03
	Cow 3	51.87

**Table 2:** Significantly different (*p* < 0.05) percentages of methylation in non-stimulated (NS) and stimulated (S) cow oocytes regarding *DNMT* family and imprinting genes. FDR = false discovery rate.

Gene	*p* Value	FDR	Difference	NS	S	% Read CGs	
						NS	S
*APEG3*	5.2618736 × 10^−5^	6.3142485 × 10^−5^	32.594048	29.62963	56.06027	40.37	69.14
*MEG3*	1.0383111 × 10^−8^	1.5574667 × 10^−8^	17.314835	23.323172	32.191566	31.12	87.15
*MEG9*	3.1143794 × 10^−4^	3.1143794 × 10^−4^	18.571428	46.951218	76.15186	39.81	93.20
*TSSC4*	0.0	0.0	41.000954	81.801994	52.26771	55.56	90.85

**Table 3:** Significantly different (*p* < 0.05) percentages of methylation in non-stimulated (NS) and stimulated (S) cow oocytes regarding *DNMT* family and imprinting CG islands. FDR = false discovery rate.

Probe	Gene	*p*-Value	FDR	Difference	NS	S	% Read CGs	
							NS	S
oe = 0.66	*MEST (PEG1)*	0.010897397	0.012454168	17.783426	97.06	77.37	23.44	86.72
oe = 0.85	*IGF2R*	5.962238 × 10^−4^	0.0011924476	11.239578	97.94	88.54	31.41	74.73
oe = 1.07	*IGF2R*	9.842796 × 10^−5^	2.6247458× 10^−4^	32.3551	97.37	65.52	60.00	64.44
oe = 0.75	*GNAS (SCG6)*	0.0020540599	0.0027387466	18.394482	97.30	81.26	15.43	70.86
oe = 0.87	*KvDMR1 ICR UMD*	0.0	0.0	45.271866	100	58.57	28.99	98.07
oe = 0.60	*IGF2*	8.9613185× 10^−4^	0.0014338109	18.31723	2.63	10.92	36.19	100
oe = 0.81	*IGF2*	0.0	0.0	45.195435	44.91	5.43	35.00	100

**Table 4:** Number of oocytes collected and sequenced for each animal from non-stimulated and stimulated cycles.

	Cow 1		Cow 2		Cow 3	
No. of oocytes	Collected	Sequenced	Collected	Sequenced	Collected	Sequenced
Non-stimulated (NS)	6	6	6	4	8	5
Stimulated (S)	11	10	9	7	29	15

## Data Availability

Data are available for review at GEO accession GSE166130: https://www.ncbi.nlm.nih.gov/geo/query/acc.cgi?acc=GSE166130, accessed on 21 November 2022. Enter token opmnmmwyhhipvux into the box.

## Supplementary Materials

The following are available online at https://www.mdpi.com/article/10.3390/ijms232416158/s1, Figure S1: Methylation in CG and non-CG context of individual oocytes; Figure S2: Methylation in CG and non-CG context of individual oocytes; Figure S3: Methylation in CGI of chromosome X versus chromosome 1 in individual oocytes; Table S1: Sequencing output of the analysed oocytes; Table S2: Hypo and hypermethylated tiles; Table S3: Hypo and hypermethylated DMRs; Table S4: Methylated and unmethylated domains; Table S5: Merged and unmerged datasets; Table S6: Enrichment analysis with merged and unmerged approaches; and Table S7: Methylation on imprinted and DNMT genes and CGI; Table S8: Data of all the sequenced libraries.
