# Supplementary figures and images for: Effect of Superovulation Treatment on Oocyte’s DNA Methylation
Source: Int J Mol Sci. 2022 Dec 18;23(24):16158. doi: 10.3390/ijms232416158 (PMC9785075; doi:10.3390/ijms232416158)

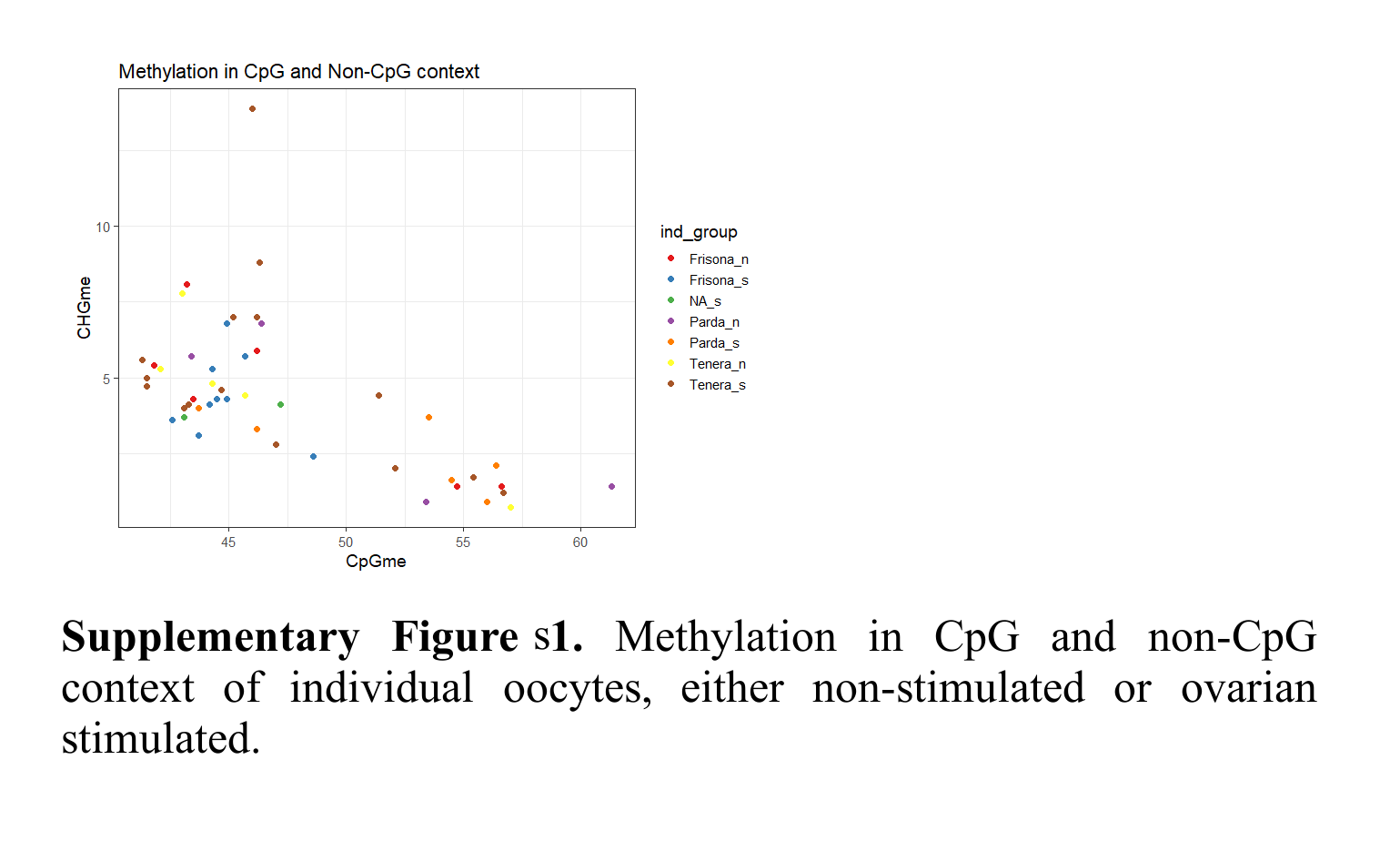

Supplement: Supplementary file 1 [file ijms-23-16158-s001.zip › Supplementary Figure S1.tif]

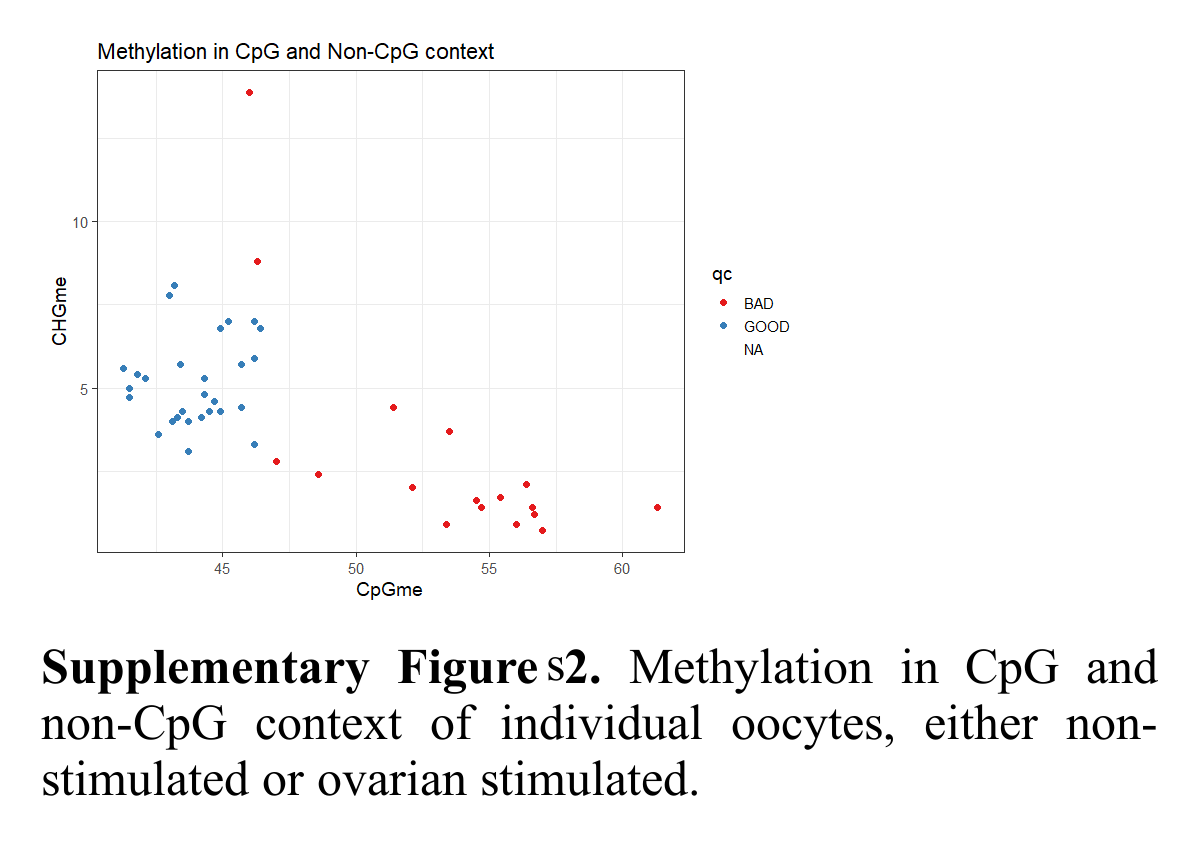

Supplement: Supplementary file 1 [file ijms-23-16158-s001.zip › Supplementary Figure S2.tif]

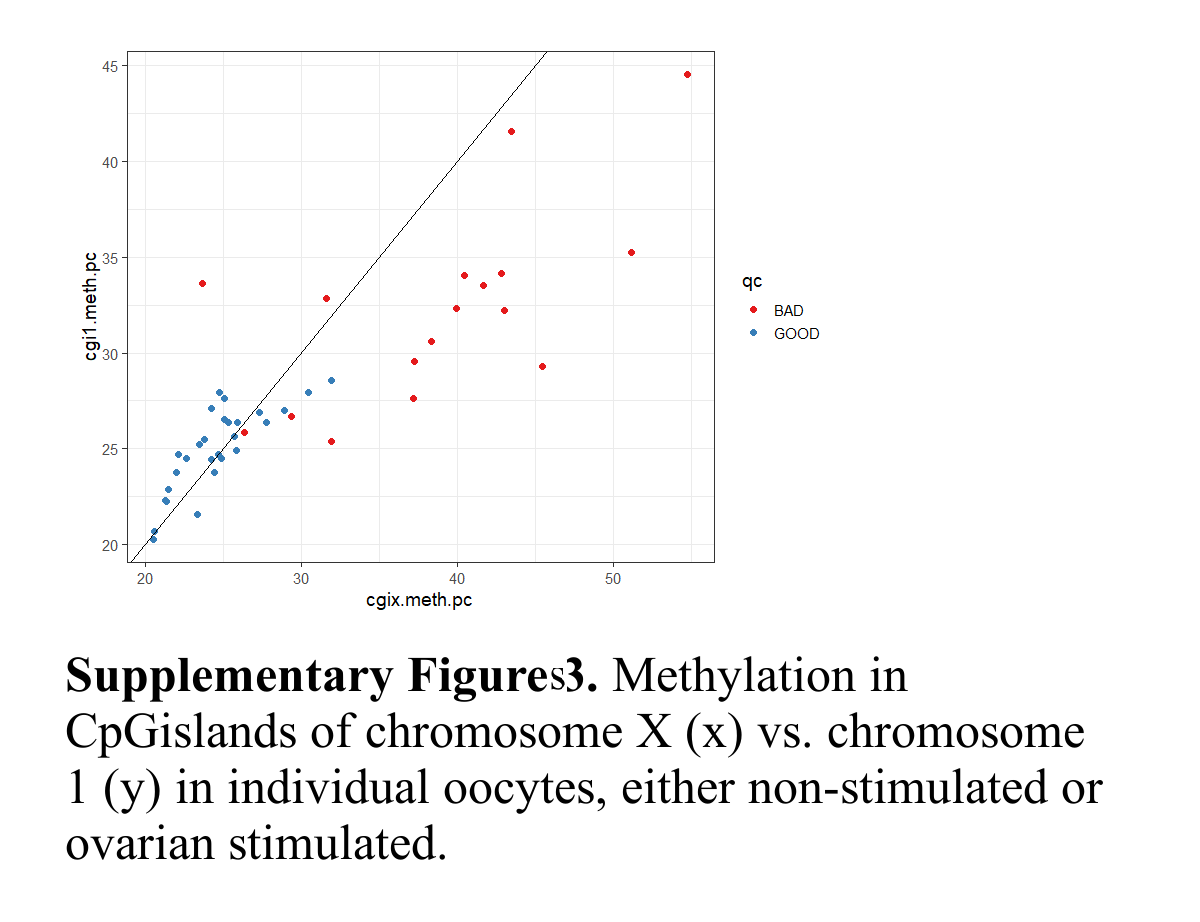

Supplement: Supplementary file 1 [file ijms-23-16158-s001.zip › Supplementary Figure S3.tif]
